# Supplementary material for: Cerebral blood flow changes during palpation of external airway structures in healthy volunteers
Source: PLoS One. 2020 Jul 27;15(7):e0236256. doi: 10.1371/journal.pone.0236256 (PMC7384655; doi:10.1371/journal.pone.0236256)
Supplement: Attachment — Submitted filename: Plos ONE Cric palpation responses 07MAY SGS.docx [file pone.0236256.s002.docx]

Dear Dr. Harris –

Thank you for reviewing our manuscript. We have revised the paper based on your peer-review feedback which we believe strengthen the manuscript. Below, you will find the reviewer feedback in bold/italics and our responses in plain text.

Sincerely,

Steven Schauer

**Comments to the Author**

1. Is the manuscript technically sound, and do the data support the conclusions?

The manuscript must describe a technically sound piece of scientific research with data that supports the conclusions. Experiments must have been conducted rigorously, with appropriate controls, replication, and sample sizes. The conclusions must be drawn appropriately based on the data presented.

Reviewer #1: Partly

Reviewer #2: Partly

2. Has the statistical analysis been performed appropriately and rigorously?

Reviewer #1: No

Reviewer #2: Yes

3. Have the authors made all data underlying the findings in their manuscript fully available?

The PLOS Data policy < Caution-http://www.plosone.org/static/policies.action#sharing >  requires authors to make all data underlying the findings described in theirmanuscript fully available without restriction, with rare exception (please refer to the Data Availability Statement in the manuscript PDF file). The data should be provided as part of the manuscript or its supporting information, or deposited to a public repository. For example, in addition to summary statistics, the data points behind means, medians and variance measures should be available. If there are restrictions on publicly sharing data—e.g. participant privacy or use of data from a third party—those must be specified.

Reviewer #1: Yes

Reviewer #2: Yes

4. Is the manuscript presented in an intelligible fashion and written in standard English?

PLOS ONE does not copyedit accepted manuscripts, so the language in submitted articles must be clear, correct, and unambiguous. Anytypographical or grammatical errors should be corrected at revision, so please note any specific errors here.

Reviewer #1: Yes

Reviewer #2: Yes

***5. Review Comments to the Author

Please use the space provided to explain your answers to the questions above. You may also include additional comments for the author, including concerns about dual publication, research ethics, or publication ethics. (Please upload your review as an attachment if it exceeds 20,000 characters)

Reviewer #1: It is a well written article. Patients requiring cricothyrotomy are usually hypoxic and this itself can raise the ICP and cause changes in the cerebral blood flow. Examining normal patients and palpating their cricothyroid has not shown a change in the cerebral blood PI and CBFV in your study. You have not used any patients with hypoxia requiring cricothyrotomy. That would have given more power to your study as you would have both cases and controls.***

Thank you for that recommendation. Since we cannot induce such injuries or physiology in healthy volunteers, we have revised our limitations to reflect this.

“Fifth, due to ethical issues, we had to use healthy individuals that were not experiencing injuries or physiology that would frequently be present after polytrauma (e.g. hypoxia, TBI, etc.). As such, our results should be considered informative for future research and hypothesis generating.”

***Reviewer #2: 1. The title is misleading***

***2. "It is unclear at this time how airway management via cricothyrotomy affects cerebral blood flow and hemodynamics. In this study we sought to determine the change in CBF during the palpation of neck landmarks in preparation for cricothyrotomy."

The study design enable us to see whether palpation evokes any CBF change in healthy probands and nothing more***

We have revised the title to the following:

Title: Cerebral Blood Flow Changes During Palpation of External Airway Structures in Healthy Volunteers

Running title: Cerebral Blood Flow During Palpation

3. Discussion

Our study has several limitations.... We do not know how the presence of a TBI would have altered our findings.

The basic data (vital data as BP CBF) of patients undergoing a cricothyrotomy are completely different than a healthy non stressed proband. You can not compare it, the only statement you can make is that CBF dos not change during palpation in healthy proband.

As noted above, we have revised the title and the limitations to better match our study design.

***Conclusion

This preliminary study suggests the palpation stage is not a potential target for interventions to avoid ICP spikes during cricothyrotomy.

This statement is not correct.***

We have removed that line from our conclusions.

6. PLOS authors have the option to publish the peer review history of their article (what does this mean? < Caution-https://journals.plos.org/plosone/s/editorial-and-peer-review-process#loc-peer-review-history > ). If published, this will include your full peer review and any attached files.


If you choose “no”, your identity will remain anonymous but your review may still be made public.


**Do you want your identity to be public for this peer review?** For information about this choice, including consent withdrawal, please see ourPrivacy Policy < Caution-https://www.plos.org/privacy-policy > .

Reviewer #1: No

Reviewer #2: No
